# From betel nuts to Cobenfy: how an ancient recreational drug gave rise to a new class of schizophrenia medications

**DOI:** 10.1017/S1092852925100424

**Published:** 2025-07-17

**Authors:** Justin Fortune Creeden, Siddharth N. Machiraju, Johansen B. Amin, Stephen M. Stahl

**Affiliations:** Department of Psychiatry, University of California San Diego, San Diego, CA, USA

**Keywords:** Arecoline, xanomeline, trospium, muscarinic agonism, parasympathomimetics, positive symptoms, negative symptoms, drug repurposing, recreational drugs, psychopharmacology

## Abstract

The term “betel” most accurately refers to the betel pepper (*Piper betle*). Confusingly, this term is also frequently used to refer to a street drug that often—but not always—includes the betel leaf as a constituent. This linguistic misdirection only intensifies with terms such as “betel nut,” which, in common usage, may refer to this same composite street drug or to a single isolated constituent of that street drug: the nut of the areca palm (*Areca catechu*), which is otherwise wholly unrelated to the betel pepper. This composite street drug, colloquially referred to as “betel” or “betel nut” or “betel quid,” is one of the most frequently used psychoactive substances in the world. It carries a cultural legacy spanning over 10,000 years and a current user base numbering in the hundreds of millions. Its primary psychoactive constituent is arecoline, a well-established parasympathomimetic agent. Early studies exploring arecoline’s ability to modulate cholinergic signaling pathways and exert therapeutic psychiatric effects on conditions such as Alzheimer’s disease were initially mired by intolerable parasympathetic side effects. Indeed, over the course of its long history, various hints regarding the therapeutic utility of arecoline have been obfuscated by a variety of challenges which have only recently been overcome. Now, developments in psychopharmacology and our growing understanding of neurochemical brain circuitry have unlocked a new mechanism of action by which arecoline-derived medications interact with dopaminergic processes to improve outcomes for schizophrenia patients. One such medication, xanomeline-trospium (Cobenfy), has emerged as the first such agent to receive U.S. Food and Drug Administration (FDA) approval for the treatment of schizophrenia and represents an entirely new class of pro-cholinergic medication within the field of psychiatry. Many in the field believe that this heralds the beginning of a new era of psychopharmacology: the era of muscarinic agonism. This article briefly described the fascinating journey from ancient betel nuts to modern muscarinic therapeutics.

## Introduction

Betel is the fourth-most consumed psychoactive substance in the world after caffeine, nicotine, and alcohol.^1^ ^,^^2^ Like Cobenfy—the novel schizophrenia medication derived, in part, from betel and composed of 2 agents: xanomeline and trospium—betel is a mixture of compounds rather than a single agent. The constituent parts that comprise betel are the nut of the areca palm (*Areca catechu*), the leaf of the betel pepper (*Piper betle*), and slaked lime (calcium hydroxide).^3^ Each component contributes to the experience of chewing betel, with parasympathomimetic arecoline from the areca nut acting as the primary psychoactive agent. As we will see, unchecked parasympathetic activation gives rise to side effects (eg, nausea, vomiting, diarrhea, cramps, high blood pressure) that decrease user satisfaction and limit therapeutic potential. For betel users, the parasympathetic effects of the areca nut may be modulated through the addition of calcium hydroxide and betel leaf. Chewing areca nut with calcium hydroxide hydrolyzes arecoline into arecaidine, which is a strong uptake inhibitor of gamma-aminobutyric acid (GABA), one of the primary inhibitory neurotransmitters in the human brain^4^ ^,^^5^; chewing it with betel leaf may further increase sympathetic nervous system activity^4^ to curb arecoline’s parasympathetic effects and minimize unpleasant adverse reactions. However, betel is a complicated topic. The term “betel” itself is ambiguous, with enormous variability in ingredients and preparations across cultural and geographic contexts. While the 3 ingredients described above—areca nut, calcium hydroxide, and betel leaf—represent the most common formulation of betel, the degree to which this particular combination actually allows users to minimize parasympathetic side effects remains unclear. We will therefore focus, for now, on the primary psychoactive component—the areca nut itself. Later, we will revisit these parasympathetic elements in the setting of emerging schizophrenia drug development strategies as we outline the events and innovations that brought us from a simple seed to an emerging class of psychiatric medication.

## Areca nut

*Areca catechu* can be found in Southeast Asia.^6^ Archeologists have evidence of *A. catechu* cultivation sites in Thailand as early as 10,000 BCE.^7^ Its seed (commonly referred to as areca nut) has since spread throughout Asia, East Africa, and the Pacific.^8^ An Indian poem from 900 BCE describes soldiers chewing areca nut,^7^ and writings from 504 BCE recount a Ceylonian (Sri Lankan) princess gifting one of her nurses an areca nut to chew.^4^ ^,^^9^ Around the first century CE, we see the areca nut described as an antidepressant in Chinese literature,^10^ with additional descriptions of areca nut as an antiparasitic agent, a digestive aid, and an aphrodisiac, appearing across a wide array of cultures and traditions.^4^ ^,^^10^ To date, more than 59 compounds have been isolated from *A. catecha*, with at least 50 isolated from the seed itself.^6^ The medicinal applications of *A. catechu* relate to these compounds’ reported ability to exert a wide range of effects on the immune, digestive, nervous, and cardiovascular systems.^6^ The chemical component arecoline, specifically, has also demonstrated antiatherosclerotic and antidiabetic properties.^7^ For our purposes, the most interesting pharmacologic effect of arecoline may be its ability to influence cognition and psychosis, discussed below. In addition to these therapeutic effects, areca nut also demonstrates pronounced deleterious effects. Beyond the adverse parasympathetic reactions previously discussed, chemical components of *A. catecha* demonstrate neurotoxic, addictive, and carcinogenic properties that may have further hampered translational efforts to develop areca-derived clinical interventions.^8^ ^,^^11^

## Arecoline

Arecoline, an alkaloid derived from areca nut, crosses the blood–brain barrier and induces a variety of subjective effects, including increased cognition, euphoria, and arousal, as well as decreased anxiety.^12^ In 1957, researchers suggested that arecoline may have utility as a treatment for patients diagnosed with schizophrenia. When patients were treated with 10 mg of subcutaneous arecoline, a small subset reported increased lucidity. When the dose was increased to 20 mg, a higher percentage of patients exhibited increasingly lucid intervals; nonverbal or catatonic patients demonstrated increased interest in their surroundings; other patients became more emotive and engaged in the psychiatric interview process.^13^ This is consistent with stimulant characteristics frequently described by betel users throughout Southeast Asia.^11^ Unfortunately, the side effects were significant and included tachycardia, increased systolic and diastolic blood pressures, tremors, and vomiting. Investigators theorized that some combination of arecoline plus the muscarinic antagonist atropine might minimize parasympathetic side effects and improve tolerance. They reasoned that methylated atropine might block the peripheral effects of arecoline, while the central muscarinic action of arecoline on the brain would remain intact and therapeutic. But it would be over 50 years before this strategy would translate to a U.S. Food and Drug Administration (FDA)-approved intervention.

## Distracted by dopamine

**Figure 1.**
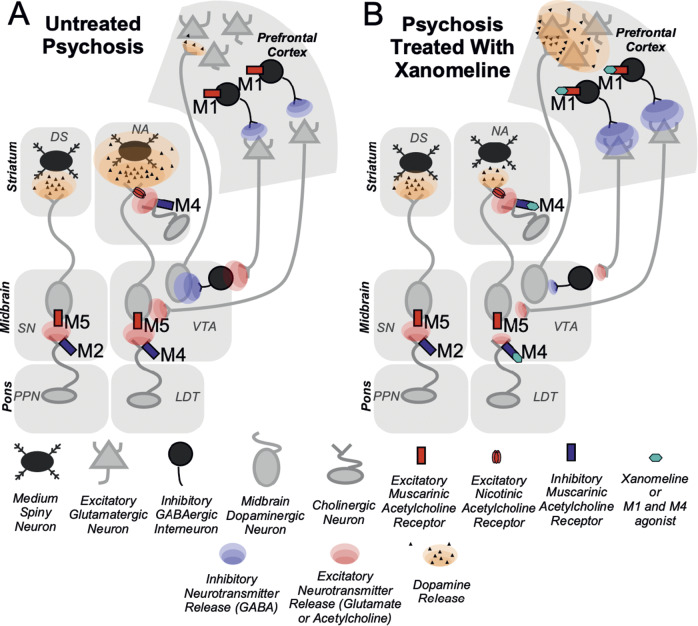
Xanomeline modulation of dopamine. (A) In psychosis, increased dopamine release in the nucleus accumbens (NA) is associated with the positive symptoms of psychosis. This is regulated by dopaminergic neurons in the ventral tegmental area (VTA) of the midbrain, which is modulated by cholinergic inputs from the laterodorsal tegmentum (LDT) as well as a combination of inhibitory and excitatory neuronal inputs from the cortex. Dopaminergic neurons from the VTA also project to the prefrontal cortex. These are regulated by a combination of inhibitory and excitatory neurons from the cortex, resulting in a low dopaminergic state that is often associated with negative symptoms in schizophrenia. (B) An M1 and M4 muscarinic acetylcholine receptor agonist (e.g., xamomeline) activates GABAergic interneurons in the prefrontal cortex via the M1 receptor. It also activates presynaptic M4 receptors in the VTA and NA. This results in synergistic reduction of dopamine release in the NA and increased dopamine release in the prefrontal cortex. Notably, the dopaminergic neurons in the substantia nigra (SN) and connections in the dorsal striatum (DS) are not affected because presynaptic receptors projecting from pedunculopontine nucleus (PPN) cholinergic neurons possess M2, not M4, muscarinic acetylcholine receptors, thereby preventing motor side effects observed in traditional antidopaminergic antipsychotics.

One reason for the delay in the establishment of arecoline and its cholinergic mechanism of action as a viable drug target might be the success of dopaminergic approaches during the first and second eras of modern psychopharmacology. Most psychiatric drugs have been “discovered” by accident, and therefore, are empiric in their origins. Our field discovers something that works, and then investigators endeavor to understand how and why. Early in the treatment of schizophrenia, medications that demonstrated the greatest efficacy were those with antidopaminergic mechanisms of action, thereby increasing pharmaceutical interest in the development of these medications. The muscarinic agonists, with their stubborn and intrusive side-effect profiles, perhaps fell out of favor as investigators and drug developers explored dopamine modulation as a potential treatment mechanism for psychiatric disorders. This conceptualization of dopamine as a major actor in psychiatric pathophysiology is sometimes referred to as “the dopamine hypothesis” or “the dopamine hypothesis of schizophrenia.”^5^ ^,^^14^ In brief, the dopamine hypothesis associates the positive symptoms of schizophrenia (e.g., psychosis) with an abundance of dopamine in the mesolimbic pathway. A simplified circuit (Figure 1) illustrates this relationship. To a lesser extent, the dopamine hypothesis also associates negative and cognitive symptoms of schizophrenia (e.g., asociality, impaired executive functioning) with a scarcity of dopamine in the mesocortical pathway. This allows us to understand why direct dopamine blockade is associated with worsened nonpositive symptoms of schizophrenia. Furthermore, when dopamine is directly blocked in the nigrostriatal pathway, patients may also experience significant motor side effects that mirror movement abnormalities observed in Parkinson’s disease. For a time, the therapeutic effects of dopamine-blocking agents overshadowed their side effects.

## A cholinergic balance to dopamine

The dopamine hypothesis remained center stage for decades. However, behind the scenes, evidence for acetylcholine’s role in schizophrenia continued to accumulate. In the late 1940s, organophosphates—chemicals that increase acetylcholine levels—entered the market as a new and affordable form of insecticides for crop protection. These chemicals, acetylcholine-esterase inhibitors that inhibit the breakdown of acetylcholine, were toxic to a wide range of insect species. But their selectivity was imprecise and affected humans as well. As their use became commonplace, agricultural workers exposed to these agents demonstrated signs and symptoms of cholinergic toxicity,^15^ ^,^^16^ the same parasympathetic effects caused by arecoline, discussed earlier. As we would expect, farmers and field workers developed muscle cramps, diarrhea, and vomiting.^15^ Moreover, they also reported puzzling psychiatric symptoms. Some even found themselves in emergency departments with acute-onset psychosis. These presentations mirrored the positive symptoms of schizophrenia so closely that many were formally diagnosed with schizoaffective disorder before the organophosphate exposure was discovered.^15^

Similarities between organophosphate and cholinergic medicament exposure led investigators to posit a relationship between acetylcholine and psychosis.^17^ Indeed, as more cases of organophosphate poisoning were reported, increased acetylcholine levels were linked to both positive and negative symptoms of schizophrenia, with individuals exposed to organophosphates also reporting significant difficulties in concentration. However, for reasons we are only just beginning to understand, the relationships between acetylcholine, positive symptoms, and negative symptoms were far from clear-cut. Other studies demonstrated that a decrease in acetylcholine elicited psychotic symptoms.^17^ ^,^^18^ Indeed, the effects of cholinergic agonists and antagonists continued to produce opposing effects in patients. Although linked, positive and negative symptoms of schizophrenia varied at different stages of the illness,^19^ with no unifying hypothesis at the time to explain a separation of the two phenomenologies. Treatments with anticholinergics to relieve motor side effects from first-generation antipsychotics were also sometimes reported to improve negative symptoms related to cognition but worsen positive symptoms.^20^ ^–^^22^ Additionally, second-generation antipsychotics with the greatest anticholinergic activity (particularly clozapine) seemed to have greater efficacy than their first-generation counterparts. Due to these observations, our field developed a renewed interest in acetylcholine as a central driver of schizophrenia.^23^ But these conflicting observations led to a big question: are schizophrenia symptoms improved by enhancing acetylcholine signaling, or are they improved by blocking acetylcholine signaling? It is no wonder that further development of cholinergic treatments for schizophrenia languished for years while this issue remained unresolved. Only later would a rational treatment development strategy emerge after investigators discovered acetylcholine’s ability to regulate dopamine release via muscarinic 1 (M1) and muscarinic 4 (M4) receptors (Figure 1A). For an in-depth pharmacological discussion, we refer readers to the excellent review by Yohn and colleagues.^17^

## Xanomeline

Based largely on the structure and pharmacology of betel’s arecoline,^24^ xanomeline was synthesized in the early 1990s as part of a drug development effort by Eli Lily and Company aimed at finding novel treatments for cognitive deficits associated with conditions like Alzheimer’s disease.^25^ In this way, xanomeline is a direct arecoline derivative.^26^ In addition to improving cognition, initial studies found that xanomeline also reduced dementia-related psychotic symptoms, which was not initially anticipated^27^ given the field’s relative lack of understanding of acetylcholine and dopamine signaling interactions at the time. Investigators wondered how and why this was occurring. After all, the drug did not directly affect dopamine. According to the widely accepted version of the dopamine hypothesis, acetylcholine would not have influenced the positive symptoms caused by high levels of dopamine in the mesolimbic pathway. Furthermore, evidence continued to mount that anticholinergic burden could be worsening cognitive and negative symptoms.^28^ ^,^^29^

Here, muscarinic acetylcholine receptors took center stage in driving the idea that this system could be a key therapeutic target. It had long been noted that administration of nonselective muscarinic antagonists could induce schizophrenia-like symptoms, implying that stimulation of these receptors might stabilize neurotransmission in affected individuals.^30^ Clinical and neuropathological studies of subjects with schizophrenia showed a reduction of M1 and M4 receptor density in key frontocortical areas involved in both the positive and cognitive symptoms, including the caudate and putamen.^31^ ^,^^32^ This reduction was also later found in the prefrontal cortex, an area associated generally with cognitive and executive function.^33^ ^,^^34^ Further, researchers began targeting the cognitive symptoms of schizophrenia with cholinesterase inhibitors, most commonly donepezil, which found success in improving working memory and executive function in multiple smaller studies.^35^ ^–^^38^

Thus, drug developers began to pivot toward models of psychosis structured around muscarinic receptor activity once it was discovered that M1 and M4 receptors regulated downstream dopamine activity (Figure 1A) and that M1 and M4 agonists reduced relevant behavioral measurements in animal models of psychosis.^17^ Thus, xanomeline, now known to be a central M1 and M4 agonist, became an increasingly attractive agent for the potential treatment of schizophrenia. However, the side effect profile remained a major challenge because xanomeline is also a muscarinic 2 (M2) and muscarinic 3 (M3) agonist. Stimulating these receptors outside of the brain creates undesirable peripheral parasympathetic side effects, specifically gastrointestinal (nausea, vomiting, diarrhea) and musculoskeletal (weakness and cramping) side effects. Notably, many patients participating in xanomeline trials dropped out due to parasympathetic overactivation.^17^ Their side effects mirrored those experienced by agricultural workers suffering from organophosphate poisoning, with both groups experiencing significant gastrointestinal and motor symptoms due to their exposure to cholinergic agents.

## Cobenfy (xanomeline + trospium)

Despite its procognitive and antipsychotic effects both in animal models and as a monotherapy in patients with Alzheimer’s disease^39^ ^–^^41^ and schizophrenia,^17^ researchers found that xanomeline as a monotherapy resulted in unacceptable peripheral cholinergic side effects,^42^ contributing to a poor tolerability profile^27^ ^,^^43^ that limited dosage options. To counteract these adverse effects, Karuna Therapeutics developed KarXT, branded as Cobenfy.^44^ ^–^^46^ Cobenfy is a combination drug comprised of xanomeline and trospium chloride (Sanctura), the latter of which is an FDA-approved antimuscarinic agent used to treat overactive bladder. Not only does trospium counteract xanomeline’s peripheral activation—especially of M2 and M3 muscarinic receptors—but it also fails to cross the blood–brain barrier, thereby mitigating anticholinergic central effects (e.g., confusion, memory impairment) while facilitating the desired procognitive and antipsychotic effects of xanomeline^46^ without blocking central M1 and M4 receptors. Tolerability of Cobenfy is best captured in multiple Phase 3 trial data, which not only replicated robust clinical efficacy in the treatment of schizophrenia but also revealed a low dropout rate secondary to adverse effects, primarily gastrointestinal, comparable to that of placebo.^47^ Although a direct head-to-head Phase 3 trial comparing the efficacy and tolerability of Cobenfy and other established antipsychotic agents has yet to occur, cross-trial data suggest that Cobenfy’s unique mechanism confers a reduced side effect burden compared to its counterparts and an effect size for clinical efficacy comparable to known D2 antagonists.^47^ ^,^^48^

The antipsychotic effects of Cobenfy can be explained by dopamine modulation achieved through distinct muscarinic receptor pathways involving complex neurocircuitry of the frontal cortex, ventral tegmental area, and nucleus accumbens^17^ ^,^^49^ (Figure 1A). The details of this neurocircuitry are discussed elsewhere,^17^ but it is worth highlighting 3 important observations. First, stimulation of postsynaptic M1 receptors in the prefrontal cortex leads to *decreased* downstream dopamine release in the nucleus accumbens (Figure 1B). This is perhaps the mechanism by which Cobenfy may improve positive symptoms in schizophrenia and Alzheimer’s disease. Second, stimulation of these same postsynaptic M1 receptors in the prefrontal cortex leads to *increased* downstream dopamine release in the prefrontal cortex (Figure 1B), a proposed mechanism by which Cobenfy may improve negative and cognitive symptoms in schizophrenia and Alzheimer’s disease. Third, simultaneous stimulation of presynaptic M4 autoreceptors in the nucleus accumbens and the ventral tegmental area leads to *decreased* dopamine release in the nucleus accumbens (Figure 1B), which explains why Cobenfy may improve positive symptoms in schizophrenia and Alzheimer’s disease.

Evidence for differential muscarinic receptor–mediated modulation of dopamine, while supported primarily by preclinical models, remains largely inferential in human subjects, given limited neuroimaging and biomarker data.^50^ ^,^^51^ However, support for these concepts is underscored by the clinical efficacy of Cobenfy demonstrated in Phase 2 and Phase 3 clinical trials. The results of these investigations support the conjecture that stimulation of central M1 and M4 receptors, while blocking peripheral M2 and M3 receptors, results in statistically significant improvements in positive and negative symptoms, while also achieving acceptable tolerability profiles compared to placebo.^46^ ^,^^52^ ^,^^53^ Furthermore, the absence of motor side effects—commonly known as extrapyramidal symptoms (e.g., dystonia, parkinsonism) associated with D2-blocking agents—further supports the notion that Cobenfy acts by cholinergic control of dopaminergic pathways as opposed to brute-force D2 blockade.^54^ ^,^^55^

As of September 26, 2024, Cobenfy is approved as a monotherapy to treat the positive symptoms of psychosis in schizophrenia. One intriguing prospect for Cobenfy is its aforementioned potential to target negative symptoms,^56^ which have largely been unaddressed by mainstream antipsychotic agents, and cognitive impairments in schizophrenia patients.^57^ Phase 3 trial data showed a statistically significant improvement in negative symptoms of schizophrenia with Cobenfy compared to placebo,^58^ ^,^^59^ though the evidence for its effects on cognitive symptoms is more nuanced. A post hoc data analysis suggests that the most pronounced improvement in cognitive function was observed in patients with greater baseline cognitive impairments as opposed to those with milder deficits.^58^ While the clinical efficacy of Cobenfy on negative symptoms is clear in replicated prospective randomized trials, both targeted studies assessing cognitive symptoms prospectively and the use of validated tools are necessary to determine the role of Cobenfy on cognition. It is possible that Cobenfy may also have utility in the treatment of psychotic and cognitive symptoms for Alzheimer’s disease—perhaps as an augmentation agent for inadequate response to traditional D2-blocking antipsychotic medications that lack concomitant central muscarinic antagonism. At the time of this writing, ongoing clinical trials are exploring these possibilities.

Cobenfy’s highly anticipated debut as an alternative to antidopaminergic agents has generated significant curiosity and excitement as psychiatry stands poised to enter a new era of schizophrenia treatment. Cobenfy has ignited a veritable “cholinergic gold rush” with more and more pro-cholinergic agents entering clinical trials. New pro-cholinergic agents under active investigation include selective M4 agonists (eg, NBI-1117568; NBI-1117569), selective M4 positive allosteric modulators (eg, emraclidine, NMRA-266), selective M1 receptor allosteric agonists (eg, NBI-1117567, ANAVEX3-71), and, like Cobenfy, additional selective M1/M4 agonists (ML-007/PAC, sebcomeline/PAC, NBI-1117570) as well as nicotinic agonists (eg, DMXB-A), and nicotinic positive allosteric modulators (eg, AVL-3288).^17^ ^,^^60^ Even familiar acetylcholinesterase inhibitors (eg, galantamine, donepezil) are under exploration as adjunctive therapy with atypical antipsychotics.^61^ ^,^^62^ It seems increasingly likely that we will soon have an entire portfolio of pro-cholinergic agents in the future.^17^ All this is due, at least in part, to that ancient betel nut.

## Conclusion

As in the case of betel, arecoline, and Cobenfy, psychiatric medications are often derived from repurposed or repositioned agents after observing—sometimes quite accidentally—therapeutic utility in other domains. While the first-generation antipsychotics of the 1950s had a profound effect on psychiatric care and marked the beginning of the psychopharmacological revolution, many of these drugs followed a similar discovery pipeline. The phenothiazines (from which the prototypic first-generation antipsychotic chlorpromazine would later be derived) were themselves derived from dyes used in the German textile industry and were first administered because of their antihistaminic actions.^63^ Monoamine oxidase inhibitors (MAOIs), the first medications specifically targeting depression, trace their origins to failed tuberculosis trials in which investigators noted remarkable mood improvements in patients treated with the prototypical MAOI isoniazid.^64^ Some aspects of lithium’s journey from a gout medication to a mood stabilizer may be attributed to anecdotal observations that drinking from lithium-containing mineral springs (such as those found in Texas’ “crazy waters”) improved psychiatric presentations^65^; certainly, valproate’s expanded indication from anticonvulsant to mood stabilizer represent another example of drug repurposing following clinical observations of psychiatric utility.^66^ ^,^^67^ Currently, there is significant interest in repurposing hallucinogenic botanicals (eg, psilocybin mushrooms) for psychiatric indications, with other plant-based medicines (“phytoceuticals”) also representing an active area of psychopharmaceutical research.^68^ While drug repurposing is not unique to the field of psychiatry, it may be disproportionately represented and inordinately reliant upon preliminary anecdotal observations and happy accidents due to logistical and ethical challenges inherent to research in this field. Limited biomarkers for psychiatric disease, difficult-to-translate preclinical models, and complex moral issues related to consenting psychiatrically unwell patients into clinical research trials are just a few examples of investigatory challenges that may contribute to the field’s exploitation of—if not reliance upon—drug repositioning pipelines. Now, it is betel’s turn to step into the (slaked) limelight as yet another drug being repositioned for psychiatric indications. Indeed, the journey from arecoline to xanomeline highlights an enduring pattern in psychopharmacology where initial serendipitous observations give rise to clinically useful translational outcomes.

As clinicians, how do we operationalize such a burgeoning schizophrenia treatment? Our opinion is that Cobenfy is positioned distinctly from its third-generation antipsychotic counterparts—such as aripiprazole and lumateperone, as well as earlier generation antipsychotics, all of which modulate dopamine activity directly—due to its novel mechanism of action directly upon the cholinergic system that underpins both its clinical benefit and tolerability profile. It is a new generation antipsychotic in a pharmacologic class by itself. The most striking difference from earlier antipsychotics is Cobenfy’s tolerability profile compared to D2 antagonists, which will potentially appeal to those patients who poorly tolerate those other agents. In terms of efficacy, it is not yet clear who might respond better to a D2 antagonist versus Cobenfy as no head-to-head trials have been conducted. Nor is it clear to what extent augmenting D2 antagonists with Cobenfy would enhance efficacy and have adequate tolerability. So far, the data suggest that effect sizes of Cobenfy in all three of its Phase 3 studies are the same magnitude as known D2 antagonists compared to placebo. With respect to cognitive symptoms, a host of current D2 antagonist antipsychotics inadvertently worsen executive function and memory due to their anticholinergic effects, as well as due to very high degrees of D2 antagonism. Whether employed as a monotherapy or as augmentation with another agent, the pro-cholinergic Cobenfy has the potential of limiting cognitive impairment while bolstering cognitive function. Further clinical trials of cognition in schizophrenia with Cobenfy will be required to determine its theoretically promising role as a procognitive agent in patients with schizophrenia.

Recent developments surrounding Cobenfy and other muscarinic agents currently under investigation may represent the beginning of what some are calling the third generation of antipsychotic pharmacology, or—to embrace more neuroscientifically grounded nomenclature, which moves away from indication-based naming conventions in favor of language that references a drug’s mechanism of action^69^—we may be at the beginning of the third era of modern psychopharmacology: the era of muscarinic agonism. At the time of this writing, xanomeline-trospium (Cobenfy) has just recently been approved by the FDA for the treatment of schizophrenia, with many more muscarinic agonists in active clinical trials (as discussed above). It has been over 70 years since the last FDA-approved psychiatric medication with a novel mechanism of action,^44^ and the field is abuzz with anticipation and hope. This new class of medications seems to sidestep many of the problematic side effects that limited the utility of previous agents while simultaneously offering distinct advantages in its potential ability to address negative symptoms. There are even indications that this new class may have procognitive effects and potential applications for Alzheimer’s disease. While still early in its life cycle, the data up to this point are compelling. By all currently available accounts, the 10,000-year journey from betel nut to a new class of psychiatric medication represents one of the most astounding drug development stories in the history of medicine.
