# From Betel Nuts to Cobenfy: How an Ancient Recreational Drug Gave Rise to a New Class of Schizophrenia Medications – ERRATUM

**DOI:** 10.1017/S1092852925100539

**Published:** 2025-09-17

**Authors:** Justin Fortune Creeden, Siddharth N. Machiraju, Johansen B. Amin, Stephen M. Stahl

Cambridge University Press apologises for an error in the originally published article. The author Siddharth Machiraju’s middle initial has been corrected from ‘M.’ to ‘N.’.

## References

[r1] Creeden, Justin Fortune, Siddharth N. Machiraju, Johansen B. Amin, and Stephen M. Stahl. “From Betel Nuts to Cobenfy: How an Ancient Recreational Drug Gave Rise to a New Class of Schizophrenia Medications.” CNS Spectrums. 2025; 30(1): e55. 10.1017/S1092852925100424. 40671308 PMC13064730

